# Survival in sinonasal and middle ear malignancies: a population-based study using the SEER 1973–2015 database

**DOI:** 10.1186/s12901-018-0061-4

**Published:** 2018-08-09

**Authors:** Mitchell R. Gore

**Affiliations:** Department of Otolaryngology, State University of New York Upstate Medical University, Physicians Office Building North, Suite 4P, 4900 Broad Road, Syracuse, NY 13215 USA

**Keywords:** Head and neck Cancer, SEER, Survival, Kaplan-Meier, Sinonasal Cancer, Middle ear Cancer

## Abstract

**Background:**

The sinuses, nasal cavity, and middle ear represent a rarer location of head and neck malignancy than more common sites such as the larynx and oral cavity. Population-based studies are a useful tool to study the demographic and treatment factors affecting survival in these malignancies.

**Methods:**

Population-based database search of the Survival, Epidemiology, and End Results (SEER) database from 1973 to 2015 for malignancies involving the nasal cavity, paranasal sinuses, and middle ear. Data were analyzed for demographics, treatment type, stage, primary site and histopathologic type. Kaplan-Meier analysis was used to assess and compare survival.

**Results:**

A total of 13,992 cases of sinonasal or middle ear malignancy were identified and analyzed. The majority of patients were between ages 50 and 80 at the time of diagnosis. Overall 5-, 10-, and 20-year survival was 45.7%, 32.2%, and 16.4%, respectively. Lymph node metastasis was reported in 4.4% of patients, while distant metastasis was present in 1.5% of cases. On univariate analysis surgical vs. nonsurgical treatment, sex, race, age at diagnosis, T stage, N stage, M stage, AJCC overall stage, primary site, tumor grade, and histopathologic subtype significantly affected survival. On multivariate analysis age, race, sex, primary site, overall AJCC stage, surgical vs. nonsurgical treatment, and T, N, and M stage remained significant predictors of overall survival.

**Conclusions:**

Malignancies of the nasal cavity, paranasal sinuses, and middle ear account for a minority of overall head and neck cancers. The overall 5-, 10-, and 20-year survival for these malignancies is relatively low. Higher T, N, M, and overall stage and higher tumor grade is associated with lower survival. Patients treated with surgery as part of the treatment regimen had higher overall survival. Demographics and primary site also significantly affect survival. Certain histopathologic subtypes were associated with poorer survival.

## Background

Malignancies of the head and neck comprise a diverse group of histopathological subtypes and tumor subsites. The majority of head and neck cancers are found in the oral cavity, oropharynx, and larynx. Malignancies of the nasal cavity, paranasal sinuses, and middle ear are far less common, with an incidence of less than 1 per 100,000 people and less than 20% of total head and neck malignancies [1, 2]. Symptoms in patients with sinonasal or middle ear cancer may be relatively innocuous, gradual in onset, and may mimic other more benign conditions, and these cancers may be discovered at a later stage relative to oral or laryngeal cancers. These factors and the relative proximity to important anterior and lateral skull base structures and neural and vascular structures may make surgical treatment more difficult, and these tumors tend to have a relatively poor survival rate.

Given the relative rarity of nasal cavity, sinus, and middle ear malignancies, this study aimed to update the population-based literature and to report the demographics, effects of treatment modality, and survival outcomes of patients treated for nasal cavity, paranasal sinus, and middle ear malignancy from the updated 1973–2015 Survival, Epidemiology, and End Results (SEER) database. The study examined the correlation between survival outcomes and the AJCC (American Joint Committee on Cancer) stage, tumor grade, tumor type, treatment type, tumor (T), nodal (N), and distant metastasis (M) stage, patient age at diagnosis, patient sex, and patient race.

## Methods

The most recent SEER database contains patient data from 1973 to 2015. Maintained by the National Cancer Institute, SEER collects data on cancer cases from various locations and sources throughout the United States, including various hospitals across many different states. The database, available at https://seer.cancer.gov/, was queried for malignant neoplasm, nose, nasal cavity, and middle ear, any subsite, any age, and was extracted using SEER*Stat version 8.3.5 (National Cancer Institute, Bethesda, Maryland) and exported into Microsoft Excel 2016 (Microsoft Corporation, Redmond, Washington) for analysis. XLstat Biomed (Addinsoft, New York City, NY/Paris, France) was used for Kaplan-Meier overall survival analysis and log-rank analysis. Statistical significance was set at 0.05. SEER data was analyzed for overall survival, patient age, sex, and race, and overall AJCC (American Joint Committee on Cancer), tumor (T), nodal (N), and distant metastasis (M) stage, surgical treatment, histopathological type, and primary tumor site.

## Results

A total of 13,992 patients with malignancies of the nasal cavity, paranasal sinuses, and middle ear were identified from the 1973–2015 SEER database. Patient demographics are summarized in Table 1. The majority of patients were > 50 years old, with age 60–64 years representing the largest group (1650/13992; 11.8%). There was a male predominance, with a 8242:5751 M:F ratio (1.4:1). Patients were 81.4% white (11,395/13992), with black patients representing 9.4% (1311/13992), other (American Indian/Alaska (AK) Native, Asian/Pacific Islander) representing 8.5% (1188/13992), and 0.7% (99/13992) unknown. Table 2 summarizes the histopathological data from the cohort. Squamous cell neoplasms (7116, 50.9%) and adenomas and adenocarcinomas (1768, 12.6%) were the most common tumor types. Table 3 summarizes the primary site data from the cohort. Nasal cavity (6455, 46.1%), maxillary sinus (4449, 31.8%), and ethmoid sinus (1205, 8.6%) were the most common primary sites.

**Table 1:** Patient demographics

	n	%
Demographics		
Total	13,992	100%
Female	5751	41.1%
Male	8242	58.9%
Black	1311	9.4%
Other (American Indian/AK Native, Asian/Pacific Islander)	1188	8.5%
Unknown	99	0.7%
White	11,395	81.4%
Age at diagnosis		
00 years	7	0.05%
01–04 years	51	0.4%
05–09 years	57	0.4%
10–14 years	64	0.5%
15–19 years	112	0.8%
20–24 years	128	0.9%
25–29 years	178	1.3%
30–34 years	280	2.0%
35–39 years	421	3.0%
40–44 years	626	4.5%
45–49 years	891	6.4%
50–54 years	1228	8.8%
55–59 years	1459	10.4%
60–64 years	1650	11.8%
65–69 years	1633	11.7%
70–74 years	1537	11.0%
75–79 years	1444	10.3%
80–84 years	1162	8.3%
85+ years	1065	7.6%

**Table 2:** Tumor histopathological types

Histopathological subtype	n	%
unspecified neoplasms	247	1.8%
epithelial neoplasms, NOS	1202	8.6%
squamous cell neoplasms	7116	50.9%
basal cell neoplasms	76	0.5%
transitional cell papillomas and carcinomas	128	0.9%
adenomas and adenocarcinomas	1768	12.6%
adnexal and skin appendage neoplasms	15	0.1%
mucoepidermoid neoplasms	190	1.4%
cystic, mucinous and serous neoplasms	94	0.7%
ductal and lobular neoplasms	27	0.2%
acinar cell neoplasms	24	0.2%
complex epithelial neoplasms	93	0.7%
paragangliomas and glomus tumors	3	0.02%
nevi and melanomas	1062	0.8%
soft tissue tumors and sarcomas, NOS	125	0.9%
fibromatous neoplasms	91	0.7%
myomatous neoplasms	388	2.8%
complex mixed and stromal neoplasms	78	0.6%
synovial-like neoplasms	9	0.06%
germ cell neoplasms	29	0.2%
trophoblastic neoplasms	1	0.007%
blood vessel tumors	52	0.4%
osseous and chondromatous neoplasms	97	0.7%
miscellaneous tumors	48	0.3%
gliomas	13	0.1
neuroepitheliomatous neoplasms	972	7.0%
meningiomas	2	0.01%
nerve sheath tumors	42	0.3%
granular cell tumors & alveolar soft part sarcoma	1	0.007%

**Table 3:** Tumor primary sites

Primary site	n	%
Nasal cavity	6455	46.1%
Middle ear	439	3.1%
Maxillary sinus	4449	31.8%
Ethmoid sinus	1205	8.6%
Frontal sinus	152	1.1%
Sphenoid sinus	442	3.2%
Overlapping lesion of accessory sinuses	281	2.0%
Accessory sinus, NOS	570	4.1%

**Fig. 1:**
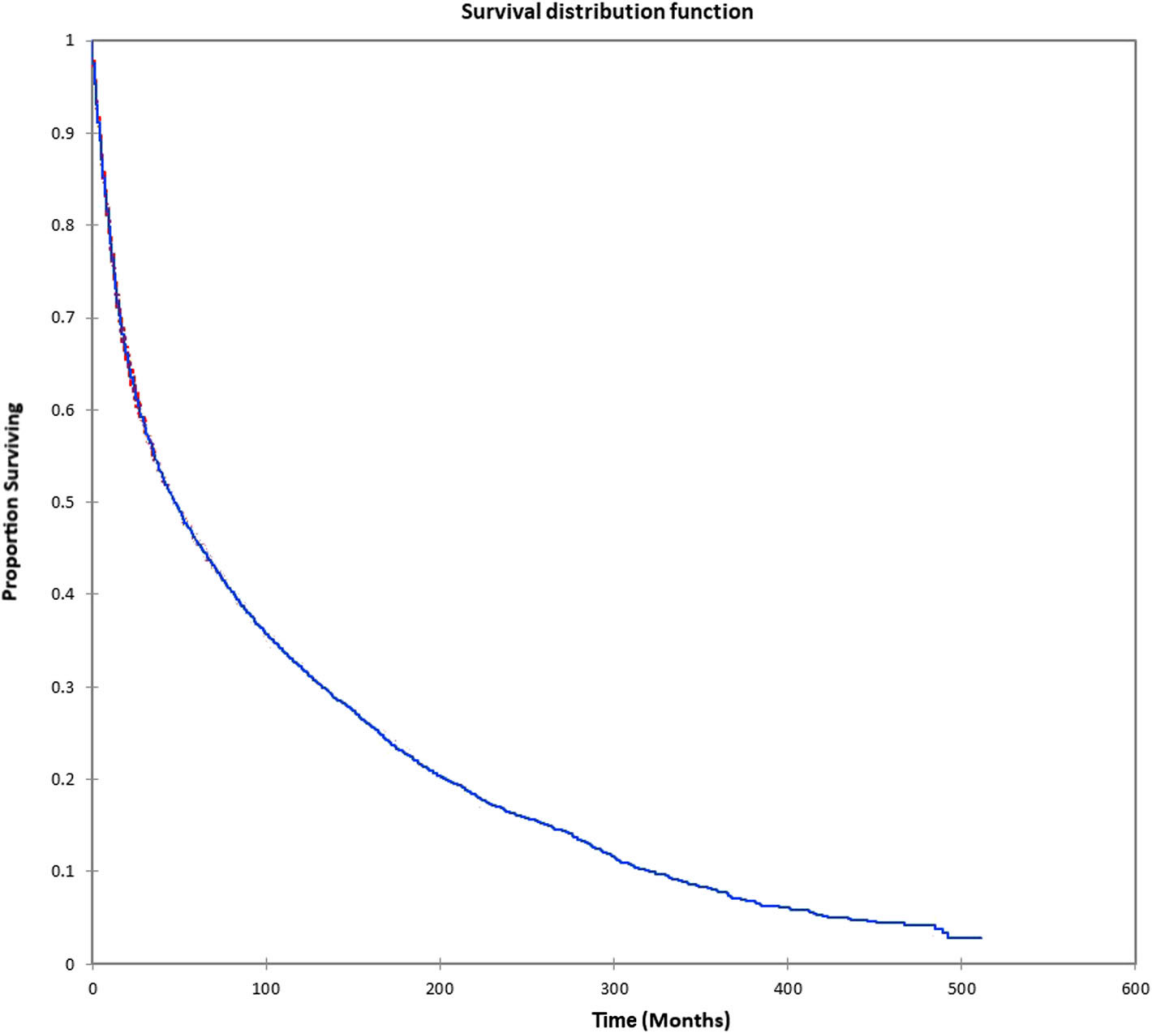
Kaplan-Meier actuarial overall survival for the entire cohort

**Fig. 2:**
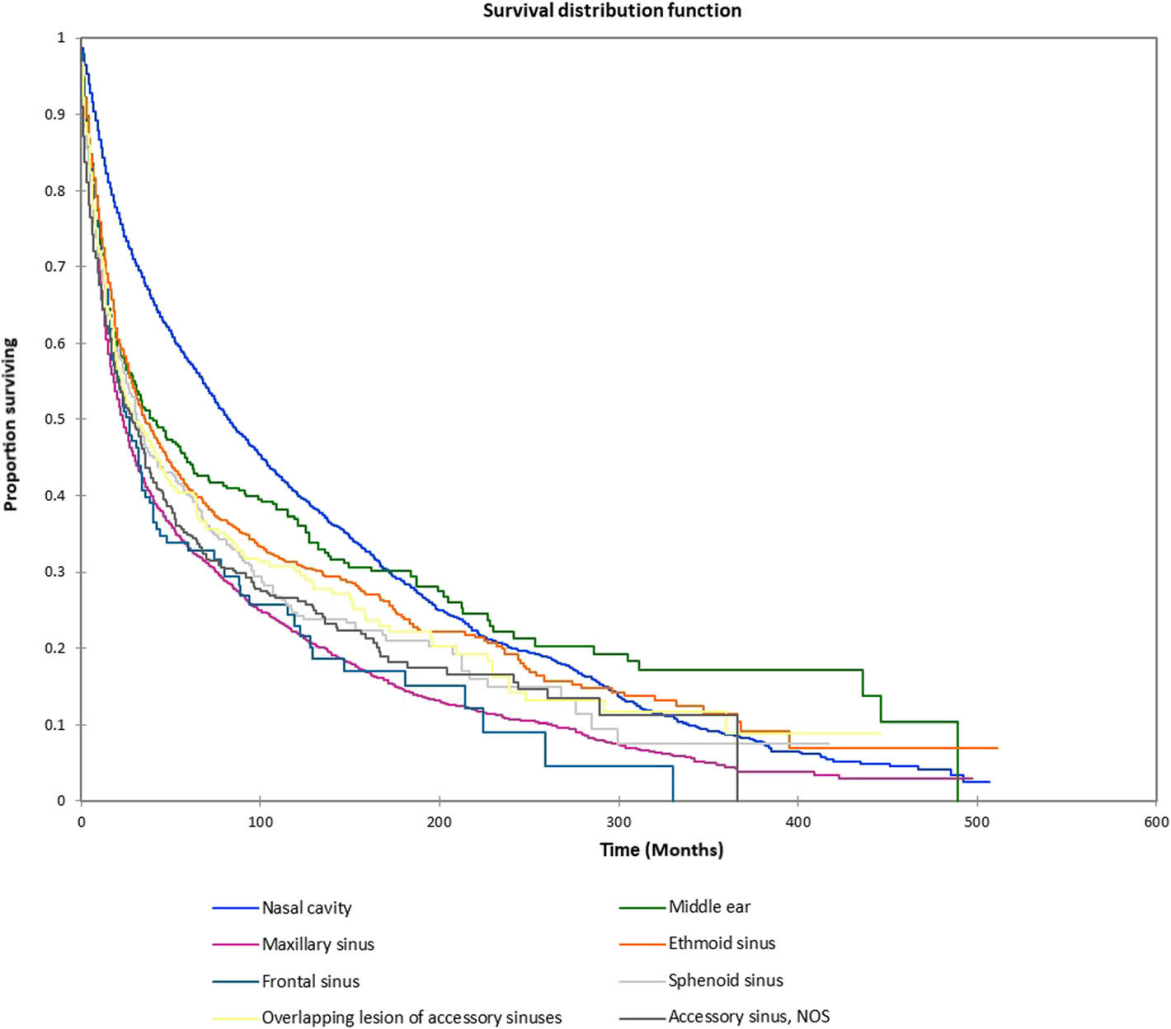
Kaplan-Meier actuarial overall survival by primary site

**Fig. 3:**
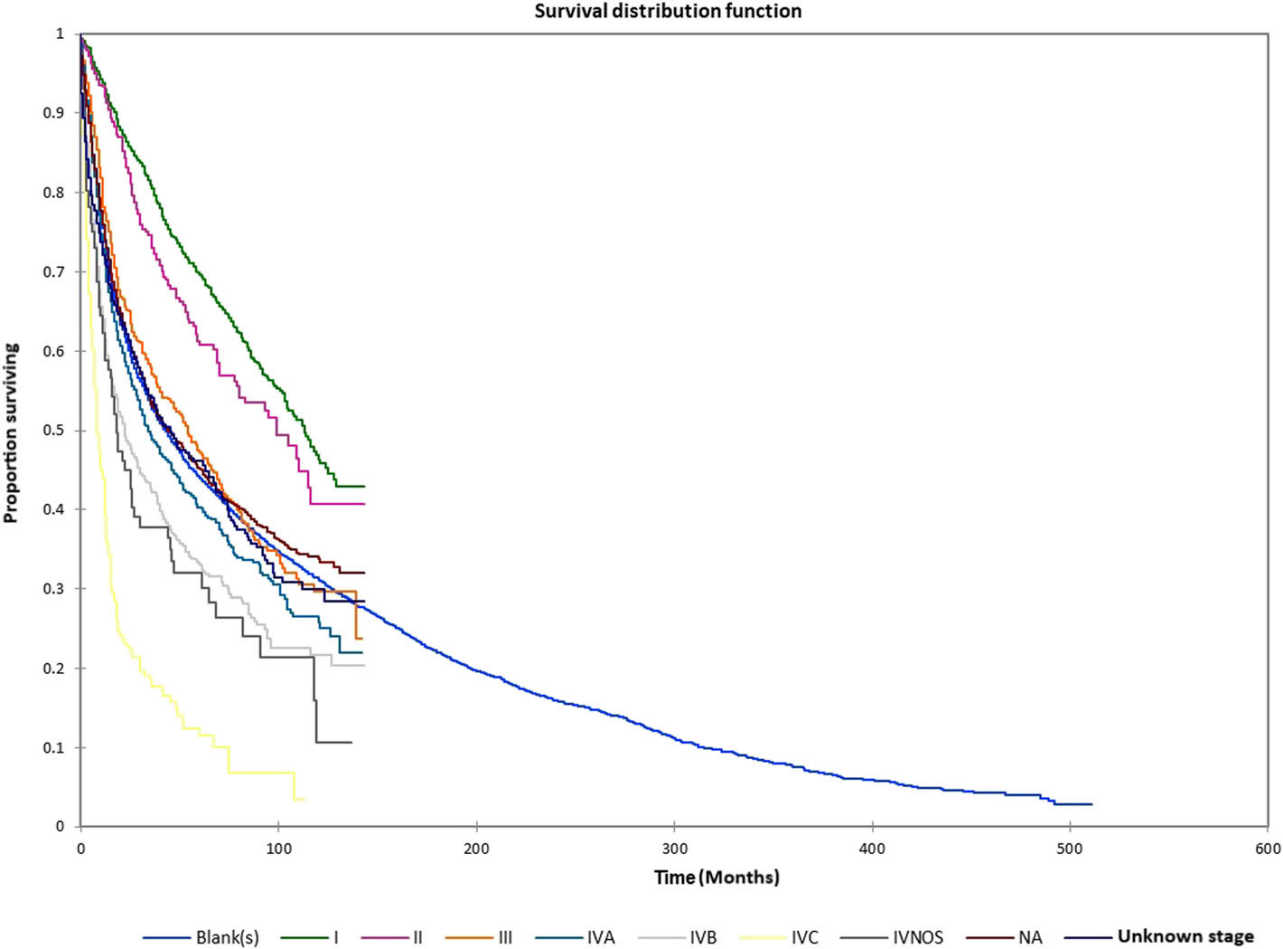
Kaplan-Meier actuarial overall survival by AJCC stage

**Fig. 4:**
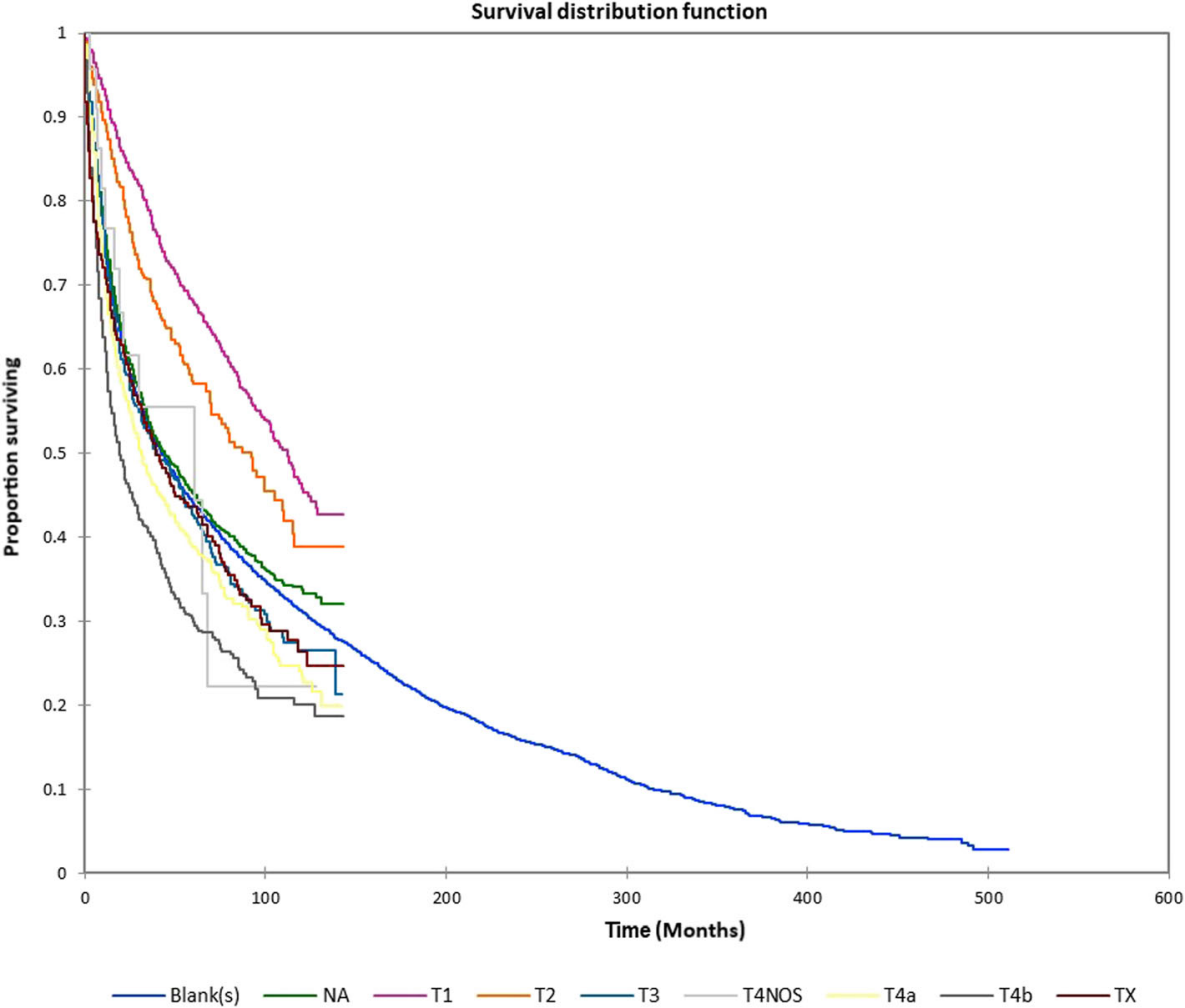
Kaplan-Meier actuarial overall survival by tumor (T) stage

**Fig. 5:**
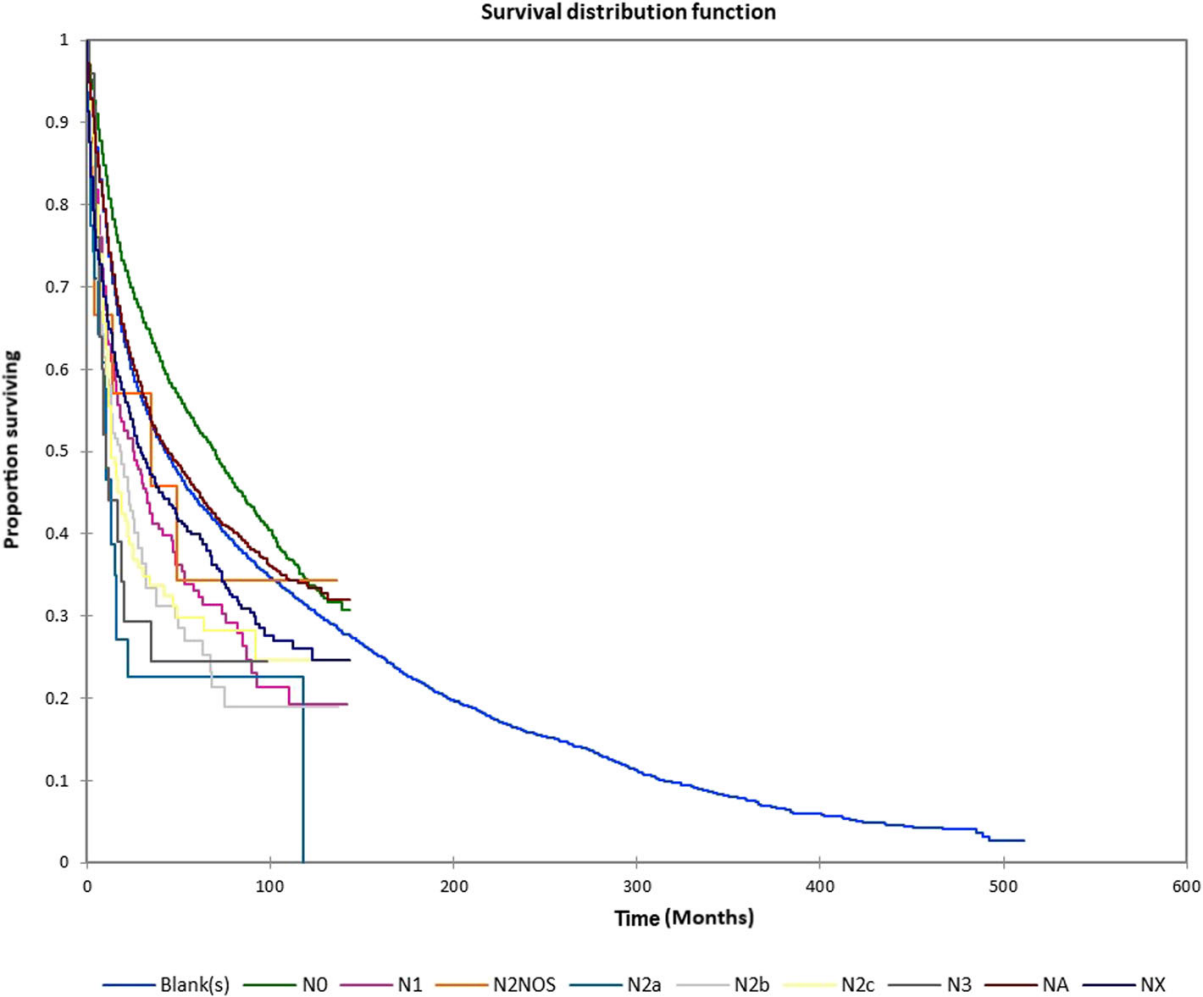
Kaplan-Meier actuarial overall survival by nodal (N) stage

**Fig. 6:**
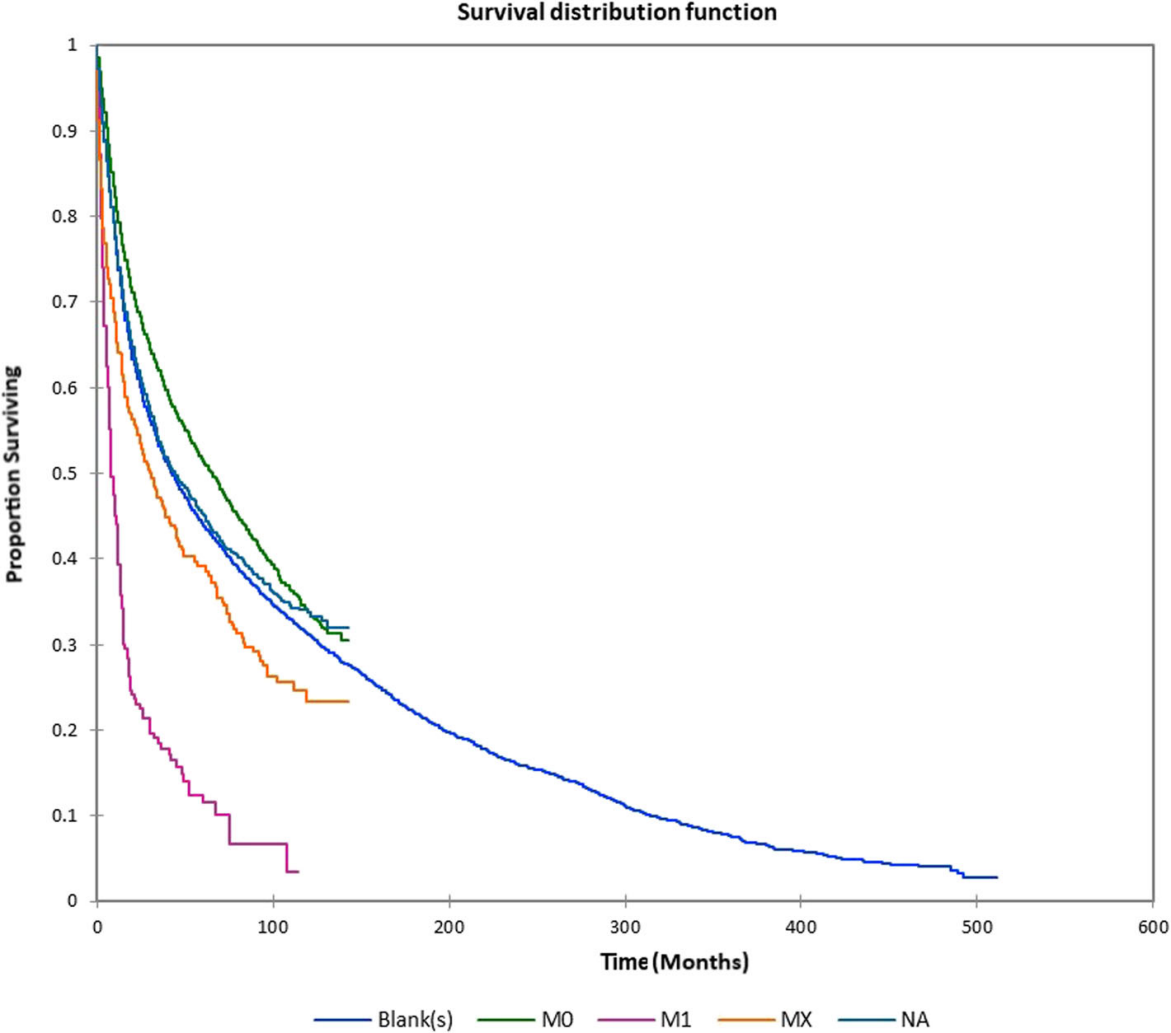
Kaplan-Meier actuarial overall survival by distant metastasis (M) stage

**Fig. 7:**
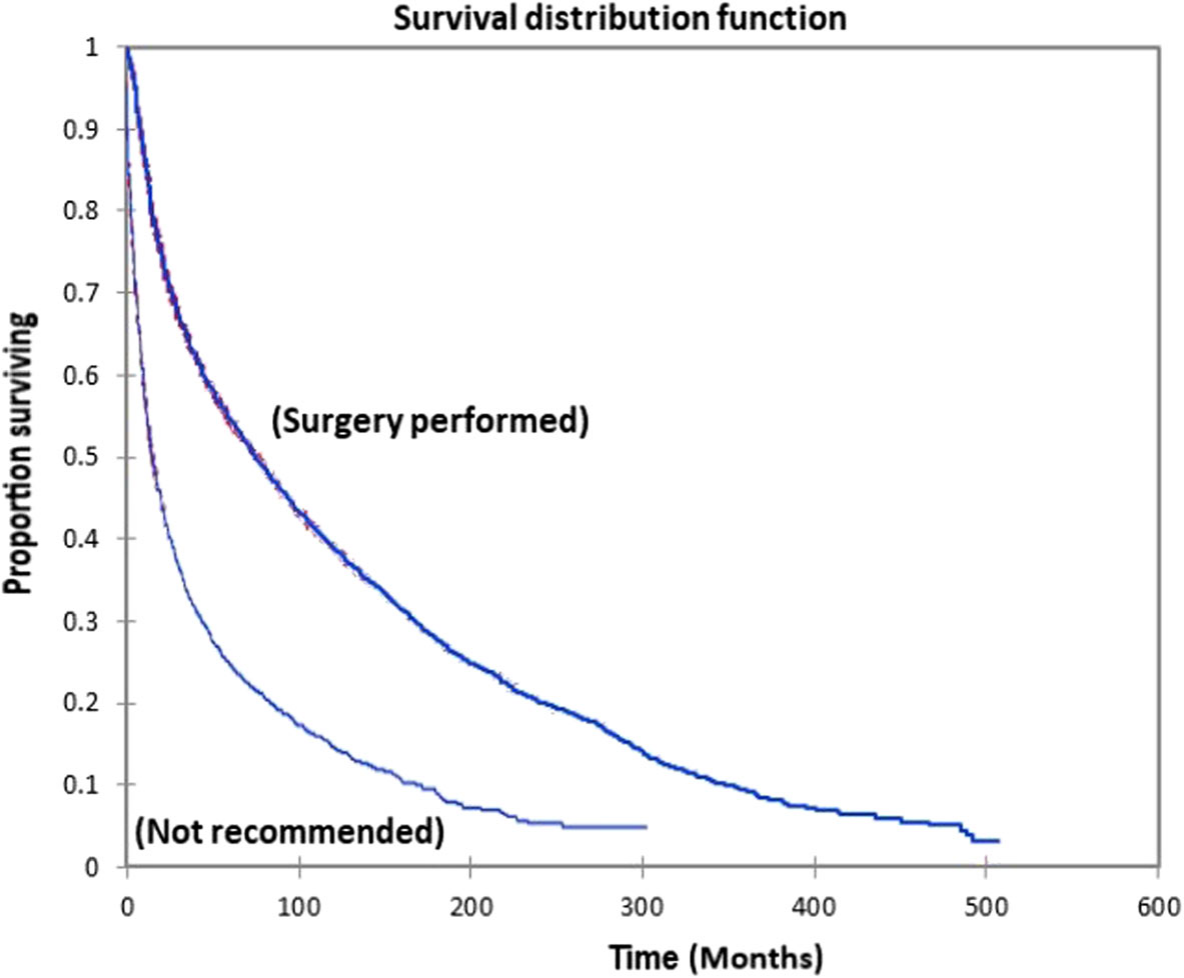
Kaplan-Meier actuarial overall survival by surgical vs. nonsurgical treatment

**Fig. 8:**
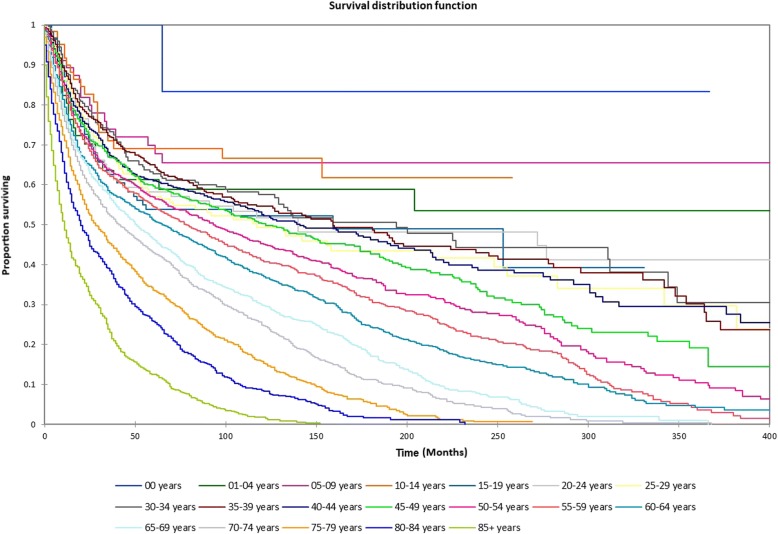
Kaplan-Meier actuarial overall survival by age at diagnosis

**Fig. 9:**
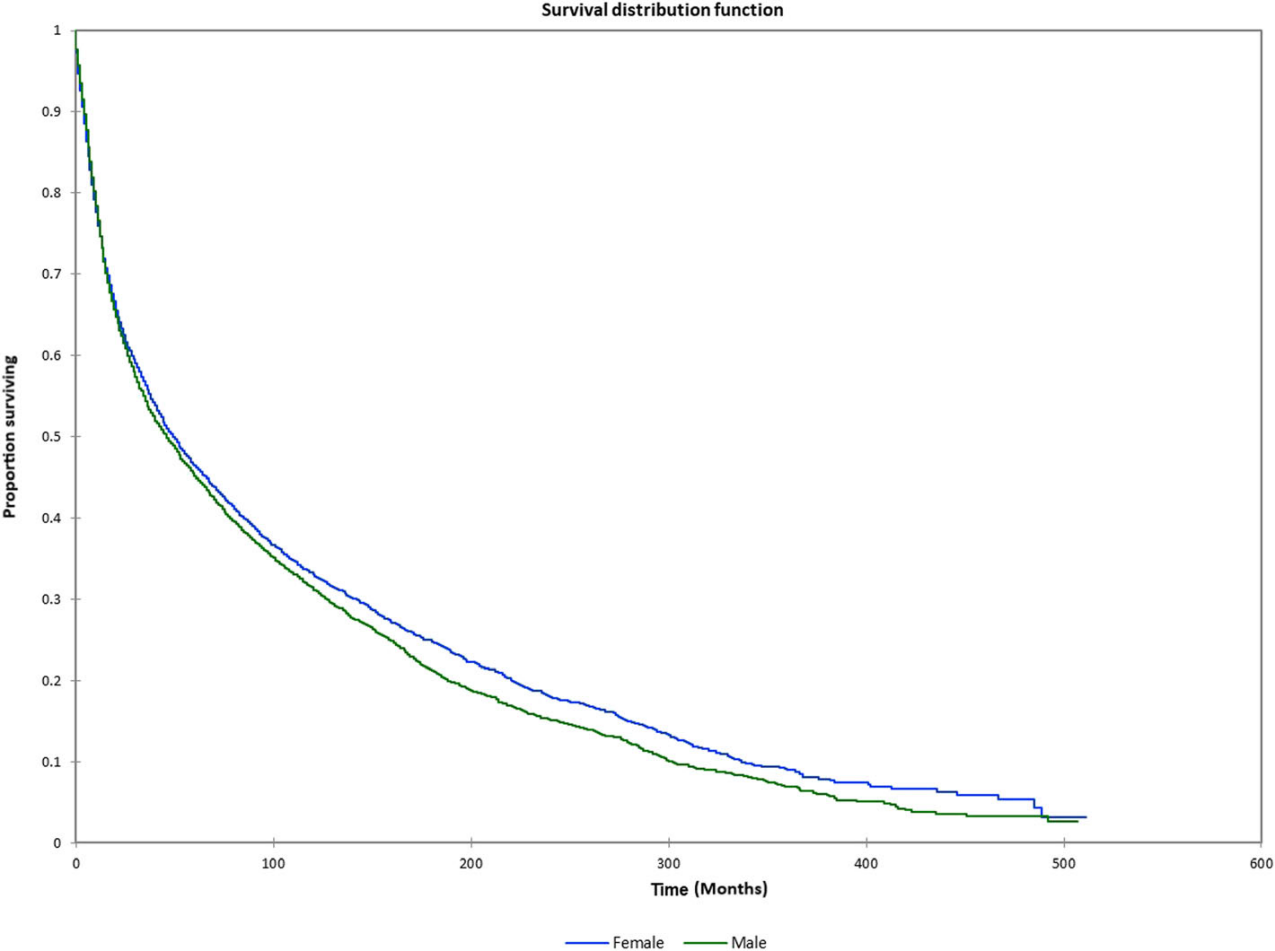
Kaplan-Meier actuarial overall survival by patient sex

**Fig. 10:**
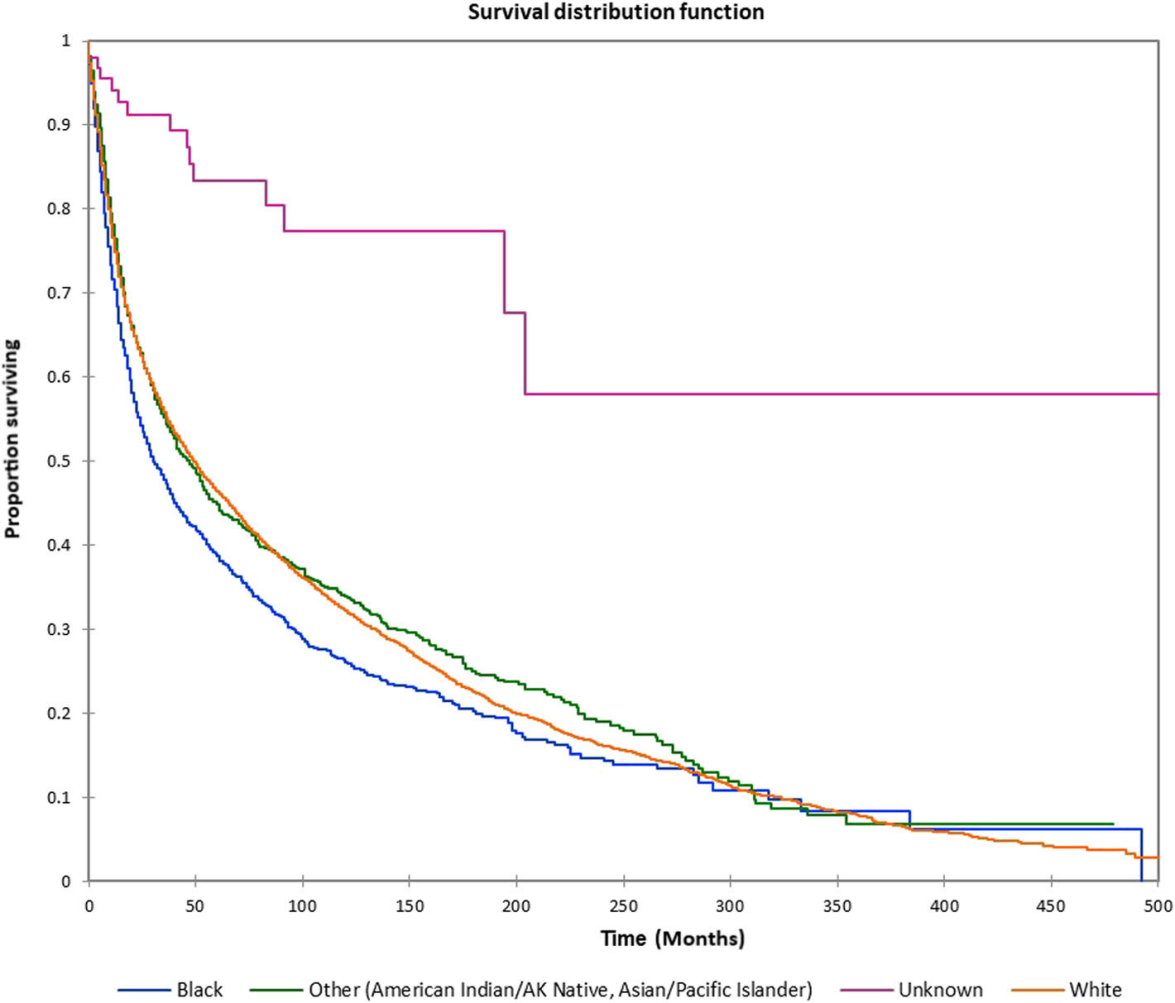
Kaplan-Meier actuarial overall survival by patient race

**Fig. 11:**
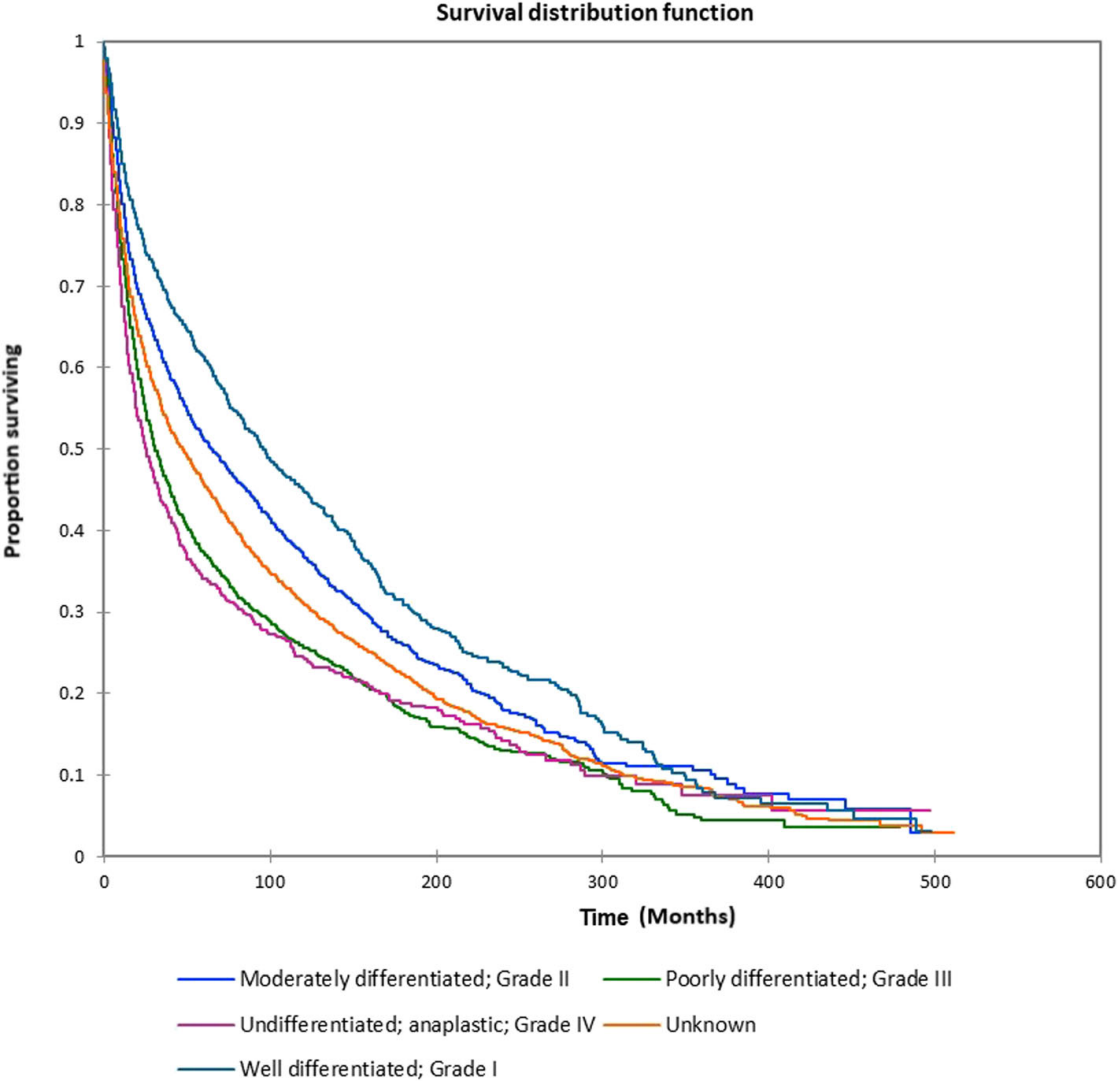
Kaplan-Meier actuarial overall survival by tumor grade

**Fig. 12:**
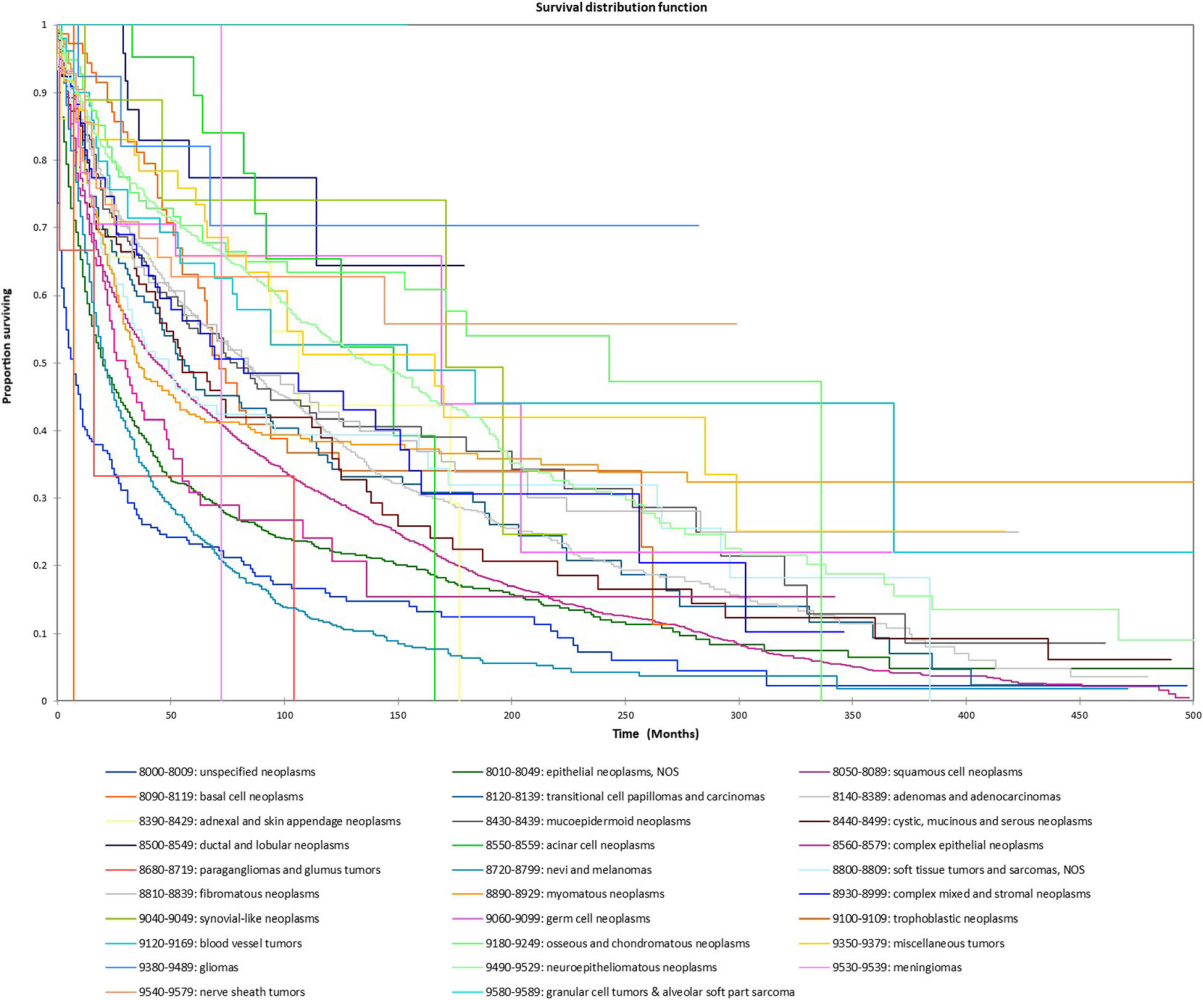
Kaplan-Meier actuarial overall survival tumor histopathological subtype

Figure 1 shows the Kaplan-Meier actuarial overall survival for the entire cohort. Five-year, 10-year, and 20-year overall survival was 45.7%, 32.2%, and 16.4%, respectively. Figure 2 shows the Kaplan-Meier overall survival by primary site. Five-, 10-, and 20-year survival was highest for nasal cavity and middle ear tumors and lowest for maxillary sinus and frontal sinus tumors (*p* < 0.001). Figure 3 illustrates the Kaplan-Meier overall survival by overall AJCC (American Joint Committee on Cancer) stage. Survival was significantly lower for Stage III and IV tumors than Stage I and II tumors (*p* < 0.0001). Figure 4 shows the Kaplan-Meier overall survival by tumor (T) stage. Survival decreased with increasing T stage (*p* < 0.0001). Figure 5 shows the Kaplan-Meier overall survival by nodal (N) stage. Survival decreased with increasing N stage (*p* < 0.0001). Figure 6 illustrates the Kaplan-Meier overall survival by M stage. Survival was significantly lower for M1 patients than M0 patients (*p* < 0.0001). Figure 7 illustrates the Kaplan-Meier overall survival for patients treated with surgery as part of their treatment regimen vs. patients for whom surgery was not recommended. Overall survival was lower for patients for whom surgery was not recommended vs. patients on whom surgery was performed (*p* < 0.0001). Figure 8 illustrates the Kaplan-Meier overall survival for patients by age at diagnosis. Survival decreased with increasing age at diagnosis (*p* < 0.0001). Figure 9 illustrates the Kaplan-Meier overall survival by sex. Survival was significantly lower for males than for females (*p* = 0.01). Figure 10 illustrates the Kaplan-Meier overall survival by patient race. Survival was significantly lower for black patients than white or other (Native American/Alaska Native/Asian/Pacific Islander) patient (*p* < 0.0001). Figure 11 illustrates the Kaplan-Meier overall survival by tumor grade. Survival decreased with increasing tumor grade (*p* < 0.0001). Figure 12 illustrates the Kaplan-Meier overall survival by tumor histopathological subtype. Survival was highest for nerve sheath tumors, gliomas, osseous and chondromatous neoplasms, germ cell neoplasms, synovial-like neoplasms, acinar cell neoplasms, adnexal and skin appendage neoplasms, and ductal and lobular neoplasms and lowest for nevi and melanomas (*p* < 0.0001). Multivariate analysis using linear regression demonstrated that age at diagnosis, race, sex, primary site, AJCC stage, surgical treatment vs. nonsurgical treatment, tumor grade, and T, N, and M stage all significantly affected survival (*p* < 0.0001, Table 4).

**Table 4:** Multivariate analysis results

Multivariate analysis variable	*p*-value
Age at diagnosis	*p* < 0.0001
Race	*p* < 0.0001
Sex	*p* < 0.0001
Primary site	*p* < 0.0001
Histological subtype	*p* = 0.2
AJCC overall stage	*p* < 0.0001
Surgical vs. nonsurgical treatment	*p* < 0.0001
T stage	*p* < 0.0001
N stage	*p* < 0.0001
M stage	*p* < 0.0001
Tumor grade	*p* < 0.0001

## Discussion

Sinonasal and middle ear malignancies account for a minority of head and neck cancers. The relative rarity of these tumors makes population-based studies a favorable method for analyzing variables and factors affecting survival in these uncommon malignancies. Dutta et al. [1] noted an incidence of sinonasal malignancy of 0.83 per 100,000 people in their SEER 1973–2011-based study. They noted a male predominance (58.6% of cases), and found that white patients comprised 81.5% of cases, while black patients accounted for 8.7%. The present study using the 1973–2015 SEER database noted a similar proportion of male patients (58.9%) and white patients (81.4%), with a slightly larger proportion of black patients (9.4%). They noted that squamous cell carcinoma was the most common sinonasal malignancy (41.9%), and that the nasal cavity was the most common primary site (45.7%). The present study found that squamous cell cancers comprised 50.9% of the cohort, and nasal cavity tumors accounted for 46.1% of patients. The Dutta study found an overall 5-year disease specific survival for all sinonasal malignancies of 53.7%, while in the present study the 5-year overall survival was only 45.7%. It is unclear what accounts for the lower overall survival in the present study relative to the Dutta study. The difference may be partially accounted for by the lower overall survival for middle ear malignancies. In their study of the 1973–2004 SEER database for middle ear malignancies Gurgel et al. [2] noted a 5-year observed survival rate of only 36.4%. In the present study the 5-year overall survival for the middle ear group was 44.4%, somewhat higher than that found in the Gurgel study but similar to the overall 5-year survival for the entire cohort in the present study (45.7%). Additionally, in the present study survival for middle ear tumors was significantly lower than that of nasal cavity tumors, but similar to or higher than paranasal sinus subsites. The higher proportion of squamous cell carcinomas in the present study relative to the Dutta study may also partially account for this difference, as the 50.9% of squamous cell neoplasms in the present study had a 5-year overall survival (45%) that was similar to that of the cohort as a whole (45.7%). Of the patients with known N and M staging in the present study, the rates of nodal metastasis (4.4%) and distant metastasis (1.5%) were relatively low. Survival rates were particularly low for patients with melanoma in the present study (5-year survival 25.1%, median survival time 51.3 months). This is consistent with previous studies [3] showing relatively dismal survival for sinonasal melanoma, with average survival on the order of 24 to 27 months.

The present study showed that on univariate analysis age at diagnosis, race, sex, primary site, histopathological subtype, AJCC overall stage, T, N, and M stage, surgical treatment vs. nonsurgical treatment, and tumor grade significantly affected survival, with all variables except histopathological subtype remaining significant on multivariate analysis. It is interesting that black patients showed significantly lower 5-, 10-, and 20-year overall survival (38.1%, 26.6%, and 14.8%, respectively) than white patients (46.4%, 32.0%, and 16.0%, respectively) and patients identified as other (46.1%, 32.5%, and 19.0%, respectively). In their 2007 study using the 1988–2002 SEER database [4] Nichols and Bhattacharyya found a similar result, noting that black patients with oral tongue and glottic squamous cell carcinoma presented with higher T and N stage, and had shorter mean overall survival. Even after controlling for stage and treatment they noted that black patients demonstrated worse survival, implying that other factors influenced survival such as extrinsic socioeconomic factors or intrinsic genetic factors, etc. In the present study race remained significant even on multivariate analysis, implying a similar effect as that seen in the Nichols study. Patel et al. [5] found a similarly lower 5-year survival for Hispanic whites and blacks (52%) vs. non-Hispanic whites (64%) in patients with sinonasal cancer in the 2000–2008 SEER database. They noted after multivariate analysis factors significantly affecting survival in addition to race were age, stage, histology, grade, comorbidity status, and standard of care, with lower stage and receiving standard of care multimodality treatment appropriate to stage being the most important prognostic factors.

The present study has limitations, including the retrospective nature of the data in the SEER database, the wide range of centers and states from which the data is compiled, and the presence of missing/unknown data for some variables such as T, N, and M stage. This makes recall and selection bias a possibility. Additionally, the inclusion of middle ear cancers (which are grouped with paranasal sinus and nasal cavity cancers in the SEER database) has the potential to add heterogeneity to the data. The fact that middle ear cancers account for only 3.1% of the cohort, and that the actuarial overall survival for the middle ear cohort is similar to the overall cohort is similar makes this less of a concern. The large number of patients in this 1973–2015 SEER cohort increases the reliability of the data, and the highly significant nature of the *p*-values noted on univariate and multivariate analysis, and the multiple studies demonstrating the utility of SEER-derived studies, makes the conclusions noted in the present study more reliable.

## Conclusions

Malignancies of the nasal cavity, paranasal sinuses, and middle ear are relatively uncommon relative to more prevalent sites such as oral cavity and larynx. The present study demonstrated that patient race, sex, and age at diagnosis all significantly affected survival, with black patients, males, and patients older than 50 demonstrating worse survival. Additionally, tumor histopathological type, primary site, grade, surgical vs. nonsurgical treatment, and AJCC, T, N, and M stage all significantly affected survival. Overall 5-, 10-, and 20-year survival is relatively low, and surgical resection when possible combined with adjuvant therapy when indicated appears to provide the best chance for survival in patients with these rare malignancies.

## Abbreviations

AJCC: American Joint Committee on Cancer
AK: Alaska
DFS: Disease-free survival
DSS: Disease-specific survival
G: Grade
M: Metastasis
N: Nodal
NA: Not available
OS: Overall survival
SEER: Surveillance, Epidemiology, and End Results
T: Tumor
